# Revealing the Bacterial Quorum-Sensing Effect on the Biofilm Formation of Diatom *Cylindrotheca* sp. Using Multimodal Imaging

**DOI:** 10.3390/microorganisms11071841

**Published:** 2023-07-20

**Authors:** Cuiyun Yang, Guojuan Song, Jiyoung Son, Logan Howard, Xiao-Ying Yu

**Affiliations:** 1 Materials Science and Technology Division, Oak Ridge National Laboratory, Oak Ridge, TN 37830, USA; 2 Laboratory for Marine Ecology and Environmental Science, Qingdao National Laboratory for Marine Science and Technology, Qingdao 266071, China; 3 Energy and Environment Directorate, Pacific Northwest National Laboratory, Richland, WA 99354, USA

**Keywords:** biofilm, diatom, N-acyl homoserine lactone, extracellular polymeric substances, morphology, ToF-SIMS

## Abstract

Diatoms contribute to carbon fixation in the oceans by photosynthesis and always form biofouling organized by extracellular polymeric substances (EPS) in the marine environment. Bacteria-produced quorum-sensing signal molecules N-acyl homoserine lactones (AHLs) were found to play an important role in the development of *Cylindrotheca* sp. in previous studies, but the EPS composition change was unclear. This study used the technology of alcian blue staining and scanning electron microscopy (SEM), confocal laser scanning microscopy (CLSM), and time-of-flight secondary ion mass spectrometry (ToF-SIMS) to directly observe the biofilm formation process. The results showed that AHLs promote the growth rates of diatoms and the EPS secretion of biofilm components. AHLs facilitated the diatom-biofilm formation by a forming process dependent on the length of carbon chains. AHLs increased the biofilm thickness and the fluorescence intensity and then altered the three-dimensional (3D) structures of the diatom-biofilm. In addition, the enhanced EPS content in the diatom-biofilm testified that AHLs aided biofilm formation. This study provides a collection of new experimental evidence of the interaction between bacteria and microalgae in fouling biofilms.

## 1. Introduction

Marine biofouling is a global issue because it causes huge economic losses and environmental pollution. Studies of fouling organisms and fouling processes are crucial for developing efficient antifouling technology [1,2]. Fouling biofilms are regarded as important constituents in the marine environment, which modulate attachment and colonization of macro-fouling organisms, such as algae, barnacles, bryozoans, and hydroids [3,4]. Bacteria and diatoms are the main components of fouling biofilms, and their interactions are paramount in biofilm communities and the biofouling process [5]. Bacteria are considered as one of the main factors that could promote the growth of diatoms and the secretion of extracellular polymeric substances (EPS) in biofilms. The latter are the dominant components in the diatom-biofilm [6,7]. In addition, earlier studies reported that the growth of algae depended on bacteria or bacterial enzymes, and algae synthesized necessary substances such as vitamin B12 via these enzymes [8,9]. Although the importance and the relevance of bacteria for diatoms have been recognized, the interactions between bacteria and diatoms are still poorly understood, especially in fouling biofilms.

Quorum sensing (QS) is the phenomenon in which bacteria regulate their behavior within the biofilm by releasing chemical signals in a cell density dependent manner [10]. It is known that the QS signals include oligopeptides amino acid cyclic thiolactone, N-acyl homoserine lactones (AHLs), furanosyl borate (autoinducer-2, AI-2), hydroxyl-palmitic acid, and many others [11]. Previous reports how show that the AHL molecules and/or AHL-producing bacterial community are dynamic during biofilm development. The dominant AHLs are found to change from short-chain to long-chain AHLs [12]. Among them, AHLs were mainly produced by Gram-negative bacteria and were widely studied in bacterial QS systems [13,14,15]. Therefore, AHL-mediated QS is extremely pertinent to marine fouling biofilms among others in the marine environment. AHLs regulated bacterial biofilm formation, stress assistance, production of toxin, and secondary metabolites in biofilm, and they mediate the interactions between different kinds of bacteria [15,16]. In addition, AHLs were reported as signals across the prokaryote-eukaryote boundary. For example, synthetic AHLs could induce the fouling alga *Enteromorpha* spores and *Chlorophyta* sp. self-aggregation [17,18]. These findings indicate that AHLs play an important role in the formation of fouling communities. At present, much attention has been drawn to the relationship between bacteria and diatoms. However, the question of how bacterial QS signal molecules AHLs mediate the growth and formation of diatom-biofilms is still under investigation.

Time-of-flight secondary ion mass spectrometry (ToF-SIMS) is an analytical method that uses a pulsed ion beam to remove molecules from sample surfaces [19,20,21,22,23,24,25]. It can detect the surface of organic samples and identify organic or inorganic layers on the surface of biofilms and directly output its molecular formula. The minimum scanning area of ToF-SIMS can reach 80 nm in diameter and depth resolution (0.1–1 nm). In addition, the three-dimensional (3D) image from ToF-SIMS would give us a clear observation of biofilm change [20]. Thus, this study utilizes the 3D image to directly observe the change of diatom-biofilm and give us EPS pictures of *Cylindrotheca* sp. under different kinds AHLs.

This study explores the possible role of AHL-based cell communication in the formation of the diatom-biofilm of *Cylindrotheca* sp. through a series of experimental assays and multimodal imaging. The effects of three kinds of AHLs on the growth and EPS production in the diatom-biofilm were investigated. Specifically, the morphology of the diatom-biofilm affected by AHLs was imaged by alcian blue staining, scanning electron microscope (SEM), confocal laser scanning microscope (CLSM), and ToF-SIMS.

## 2. Materials and Methods

### 2.1. Algal Cultures and Chemicals

*Cylindrotheca* sp. was isolated and purified from a fouling biofilm in April 2012,Yantai Coast (37°30′48″ N, 121°26′41″ E), northern Yellow Sea China. The diatom was treated with 300 mg/L penicillin, 100 mg/L streptomycin and 100 mg/L gentamycin for 36 h. Afterwards, single cells were isolated by micropipetting and transferred to sterile f/2 culture medium [26], and the components are shown in Table S1. Then, they were cultured to the exponential phase for experiments. The diatom *Cylindrotheca* sp. was stored in the Key Laboratory of Coastal Biology and Biological Research Utilization, Yantai Institute of Coastal Zone Research, Chinese Academy of Sciences (CAS). The strain is a unicellular benthic diatom, easily attached to the surface of solids and cultured with f/2 medium in a light incubator, at light intensity of 48 μmol photons m^−2^s^−1^ and 20 °C with a 12 h light/12 h dark cycle. Bacterial quroum-sensing molecule AHLs were chosen based on the different carbon-chain length: namely, C4-HSL, C8-HSL, and C12-HSL were synthesized in Yantai Institute of Coastal Zone Research, CAS, and the structure was confirmed by ^13^C NMR and ^1^H NMR and the purity was detected by GC-MS (Figure S1). The purities of the AHLs were higher than 95%. All other chemicals were analytical grade or higher. They were purchased from commercial sources and were used as received.

### 2.2. The Growth and EPS of the Diatom-Biofilm Assay

To evaluate the effects of AHLs on the growth and formation of the diatom-biofilm of *Cylindrotheca* sp., the attached-diatom cellular Chlorophyll a (Chl.a), cell numbers, and EPS including protein (PN) and polysaccharide (PS), were determined, which were conducted in 24-well polystyrene plates. The total volume of every well was 2 mL including the diatom culture medium and AHLs. The initial cell density was 1~2 × 10^5^ cells/mL and the concentrations of AHLs were prepared as 0, 0.1, 1 and 10 mg/L. DMSO was used as the diluent to dissolve AHLs. DMSO was also used as the solvent control. The control and treatment groups were at least six replicates for experimental data analysis. Because AHLs were easily degraded in f/2 medium in seawater, the method of Tait and Havenhand was employed to maintain the desired concentration of AHLs during the long-term experiment [27], and the percent degradation of AHLs was calculated as 2.57 ± 0.01% per h for C4-HSL, 2.72 ± 0.04% per hour for C8-HSL, and 3.38 ± 0.22% per h for C12-HSL with a reference to the established calibration curve. Using these values, AHLs were replenished in the medium every 24 h. After the 24-well plate was incubated for 15 days, the supernatant in 24-well plate was removed. Then, the wells were washed twice with phosphate buffer (PBS, pH 7.8) to remove the unattached cells. Other attached cells in the 24-well plate were regarded as the biofilm-forming cells, and they were used for analysis of cell numbers, Chl.a, and EPS contents.

A portion of the diatom-biofilm samples were fixed with 2% glutaraldehyde for 1.5 h, and then the cell numbers were determined using a hemocytometer and microscopy (Olympus BX51, Shinjuku City, Japan). Another portion of the samples was used to determine Chl.a contents according to a reported method [28]. Briefly, the diatom-biofilm was immersed in 95% ethanol over night at 4 °C. The pigment contents were calculated using the following equations: Chl.a = 13.95A_665_ − 6.88A_649_. The rest of samples were used to determine the EPS contents. The total EPS of every well in the 24-well plate was extracted with 2 mL sterilized distilled water, 30 °C water bath for 1 h, and centrifuged at 5000× *g* for 10 min. Then, the PN and PS contents in the cell-free supernatant were analyzed in accordance to the colorimetric methods described by Cohen and Walt [29] and Masuko et al. [30], respectively.

### 2.3. The Morphology of Diatom-Biofilm Assay

The diatom-biofilm was stained with alcian blue and observed with SEM, CLSM, and ToF-SIMS. A similar experimental procedure was used to prepare samples. The diatoms were cultured in the 24-well plate and the concentration of AHLs was initially set as 10 mg/L. After 15 days’ incubation, the supernatant was removed and the diatom-biofilm was washed twice with PBS (pH 7.8), fixed with 2% glutaraldehyde for 1.5 h, and then 1 mL alcian blue (1% *w*/*v*) in 3% acetic acid was added for 30 min. Finally, the alcian blue was washed twice with PBS (pH 7.8) for photos [31].

For SEM, the study was conducted in 12-well plates and each well contains 1 × 1 cm^2^ glass slides. The total volume of each well was 4 mL including 10 mg/L AHLs, and the diatom density was the same as the above. The glass slides were ultrasonically washed three times in distilled water and sterilized for the following experiment. After 15 days incubation, the covered diatom-biofilm slides were taken out and washed genlty three times with sterile seawater to remove unattached cells and then fixed with 4% formaldehyde for 1 h, followed by gradient washing in a series of aqueous solutions of 25%, 50%, 75%, and 95% ethanol for 15 min, respectively. The slides were dried and kept in a dessicator overnight before SEM analysis (Hitachi S-4800, Tokyo, Japan).

CLSM (FluoView FV1000, Olympus, Japan) was used to determined the three-dimensional (3D) structure of the diatom-biofilm of *Cylindrotheca* sp. The diatoms were cultured in the 24-well special-ordered plate for direct CLSM scannning and the concentration of AHLs was set as 10 mg/L initially. After 15 days’ incubation, the supernatant was removed and the diatom-biofilm was washed twice with PBS (pH 7.8), fixed with 2% glutaraldehyde for 1.5 h, and then stained with 50 μg/mL concanavalin A (ConA) conjugated to fluorescein isothiocyanate (FITC) for probing EPS in the diatom-biofilm for 30 min in 4 °C refrigerator. Then, the dye was washed twice with PBS (pH 7.8) and the diatom-biofilm was scanned with CLSM. The excitation wavelength was 488 nm and the emission wavelength was 515 nm. The scanning step distance was set as 1 μm to determine the thickness of the diatom-biofilm. CLSM images were generated by using the software FV10-ASW (version 4.0, https://www.olympus-lifescience.com/en/downloads/detail-iframe/?0[downloads][id]=847249651, accessed on 2 March 2019)) and the fluorescence intensity of the biofilm was also analyzed [32]. A series of scanning photos were used to determine the thickness of the biofilm; images with the strongest fluorescence intensity were randomly selected from at least twenty regions to calculate the values of fluorescence intensity of a sample. Finally, the mean values were used to represent the fluorescence intensity of each group.

ToF-SIMS, a powerful imaging mass spectrometry technique was used to directly acquire chemical mapping and molecular characteriaziton of the total EPS of diatom-biofilms. Similar to the above experiment, the diatom-biofilms were cultured in the 24-well plates and each well containing 2 mL cultures and 10 mg/L C12-HSL. After 15 days’ incubation, the cultures of 2 mL were gently centrifuged under 5000× *g* for 10 min and the supernatant removed. Cells were washed with deionized water (DI) three times and re-suspended in 1 mL DI, then 20 µL of solution containing diatom cells was dripped on a cleaned silicon (Si) wafer (10 mm × 10 mm) [22,23,24]. The f/2 medium, f/2 medium and C12-HSL, and diatom cells without AHLs were used as the control group to show the effect of C12-HSL on the EPS contents of diatom-biofilms.

After nitrogen drying in the hood, samples were analyzed with ToF-SIMS (ToF-SIMS V, IONTOF GmbH, Münster, Germany). During static ToF-SIMS analysis, the 25 keV Bi_3_^+^ primary cluster ion beam wasb used with a current of 0.56 pA at 10 kHz pulse energy. The main chamber pressure was maintained at ~8 × 10^−9^ mbar throughout analysis. High mass resolution mass spectra were acquired by rastering the primary ion beam over an area of 500 × 500 µm^2^ for 100 scans with 128 × 128-pixel resolution [22,33,34]. ToF-SIMS data were analyzed using the IONTOF Surface Lab 6.0 software and at least four positive and negative data points for each sample were collected. The ions used for mass calibration in the positive mode included CH_4_N^+^ (*m/z^+^* 30), C_3_H_9_N_2_^+^ (*m/z^+^* 73), CH_9_N_4_^+^ (*m/z^+^* 77), and C_19_H_39_O_2_^+^ (*m/z^+^* 299). The ions used for mass calibration in the negative mode were C^−^ (*m/z*^−^ 12), C_4_H_7_O^−^ (*m/z*^−^ 71), and C_20_H_39_O_2_^−^ (*m/z*^−^ 311). The mass calibrated data were exported to Origin Pro (2019b) for plotting.

### 2.4. Data Analysis

All the data except ToF-SIMS data shown in the study were the means ± SE of at least six replicates and were evaluated by using one-way analysis of variance (ANOVA) followed by the least significant difference test, *p* < 0.01 and *p* < 0.05 (Origin 7.5 for Windows). ToF-SIMS data were analyzed with the IONTOF Surface Lab software 6.0, and at least five positive and negative points were analyzed [21]. The ions intensity representing the EPS of diatom-biofilms were used to reconstruct two dimensional (2D) images. The data were mass calibrated in the SurfaceLab software version 6 before the results were exported and plotted in Igor 8.0 [23,25,35].

## 3. Results

### 3.1. Effects of AHLs on the Growth of the Diatom-Biofilm

**Figure 1:**
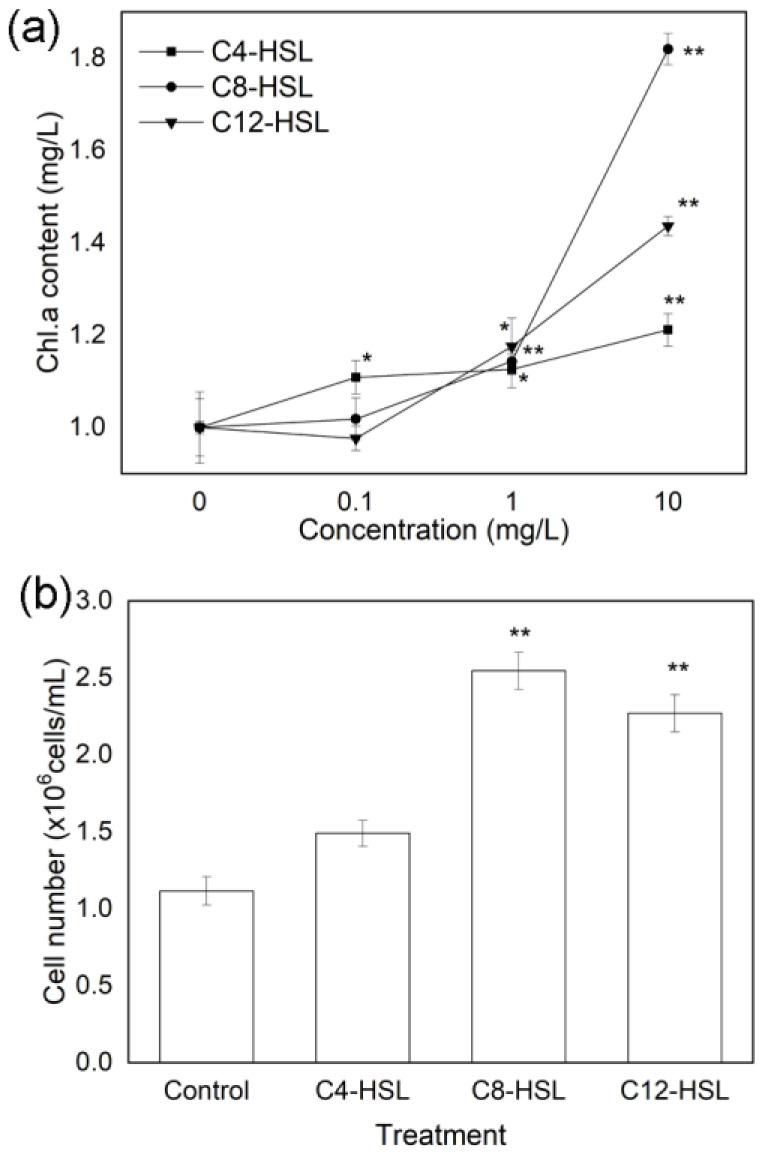
Effects of AHLs on the biomass of diatom-biofilm (*Cylindrotheca* sp.). All error bars indicate SE of the means. * (*p* < 0.05) and ** (*p* < 0.01) indicate significant differences compared with the corresponding control without AHLs. (**a**) Chlorophyll a (Chl.a); (**b**) algal cell numbers at the exposure concentration of 10 mg/L AHLs.

AHLs exposure shows a positive effect on the growth of the diatom *Cylindrotheca* sp. (Figure 1). The total contents of Chl.a show a gradual increasing trend with the increase in the AHLs concentrations. At the higher concentration of AHLs (10 mg/L), three kinds of AHLs (i.e., C4-HSL, C8-HSL and C12-HSL) significantly promote Chl.a contents in the diatom-biofilm, and the values are in a rising order, namely 1.21, 1.82, and 1.44 times of the control. In addition, C4-HSL of 0.1 mg/L and three kinds of AHLs at the concentration of 1 mg/L increase the contents of Chl.a in the diatom-biofilm in comparison with that of the control (Figure 1a). By calculating the numbers of diatom cells with optical microscopy, AHLs (C8-HSL and C12-HSL) of 10 mg/L obviously increase the cell numbers of the diatom-biofilm and the values are 2.28 and 2.04 times of the control, respectively (Figure 1b).

### 3.2. Effects of AHLs on the EPS Contents in the Diatom-Biofilm

**Figure 2:**
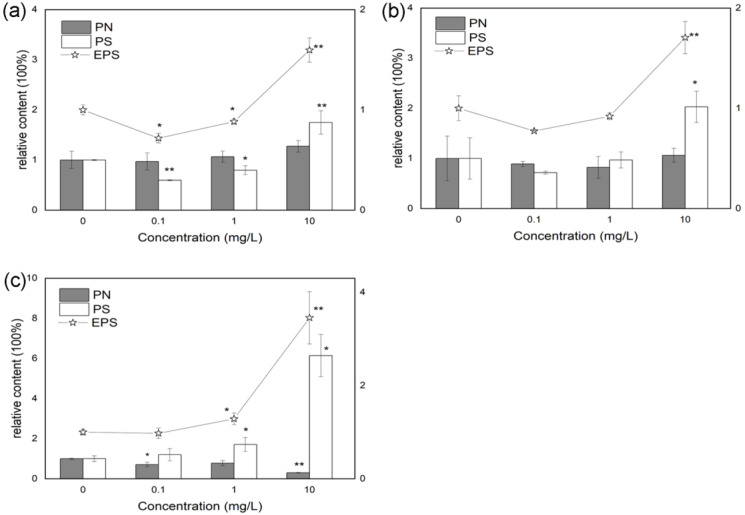
Effects of AHLs on protein (PN), polysaccharide (PS), and the total extracellular polymeric substance (EPS) contents in the diatom-biofilm (*Cylindrotheca* sp.). All error bars indicate SE of the means. * (*p* < 0.05) and ** (*p* < 0.01) indicate significant differences compared with the corresponding control without AHLs. (**a**) C4-HLS; (**b**) C8-HSL; (**c**) C12-HSL.

Figure 2 depicts the effects of AHLs on the contents of EPS including PN and PS in the diatom-biofilm (*Cylindrotheca* sp.). The PS contents are decreased upon C4-HSL of 0.1 and 1 mg/L exposure and the values are 59.69% and 79.72% of the control (Figure 2a). However, when the concentration of C4-HSL reaches 10 mg/L, the PS content is significantly increased with the value of 1.75 times of the control. The PN contents show no variations between the treatment groups of C4-HSL and the control. Therefore, the total EPS contents decrease corresponding to the exposure concentrations of 0.1 and 1 mg/L, and they increase at the concentration of 10 mg/L C4-HSL (Figure 2a). For C8-HSL, the PS and total EPS contents increase when the exposure concentration reaches 10 mg/L (Figure 2b). C12-HSL of 1 and 10 mg/L increase the PS contents in the diatom-biofilm and the values are 1.71 and 6.15 times of the control. However, C12-HSL shows a negative effect on the PN contents when the exposure concentrations are 0.1 and 10 mg/L. The total EPS contents are significantly increased when the diatom is exposed to 1 and 10 mg/L of C12-HSL (Figure 2c).

The ratios of EPS and Chl.a, PN, and PS in the diatom-biofilm are calculated and listed in Table 1. The values of EPS/Chl.a are increased when the diatom-biofilm is exposed to 10 mg/L C4-HSL and C12-HSL, and the value of C12-HSL is higher than that of C4-HSL. By contrast, C8-HSL of 10 mg/L expsoure shows no change in the value of EPS/Chl.a compared with the control. The PN/PS results show that C8-HSL and C12-HSL have decreased ratios and that the treatment group of C4-HSL has no significant difference between other treatment groups and the control.

### 3.3. Effects of AHLs on the Morphology of the Diatom-Biofilm

**Figure 3:**
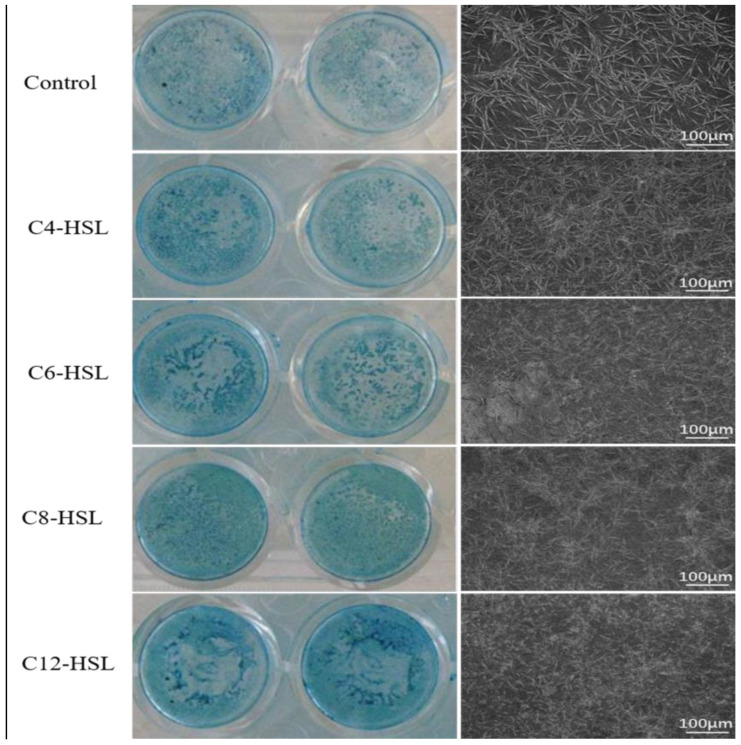
Effects of AHLs on the morphology of diatom-biofilm (*Cylindrotheca* sp.). The treatment concentration of AHLs is 10 mg/L. The left images show the results of alcian blue staining and the right ones are taken by SEM. The bar represents 100 μm.

Figure 3 depicts the effects of three kinds of AHLs on the morphology of the diatom-biofilm of *Cylindrotheca* sp. by alcian blue staining assay and SEM imaging. It is found that the biofilm formation is accompanied by much denser alcian blue staining, and the long carbon-chain signal molecule AHLs are more likely to promote the formation of the diatom-biofilm. The SEM images show that the diatom cells in the biofilm of all the treatment groups are much tighter than that of the control.

Biofilms include cells and EPS (PN and PS) secreted by bacterial cells. In Figure 3, alcian blue staining shows that the PN and PS contents increase with the long carbon-chain AHLs. The SEM images show more dense cell culture in AHL-treated groups. This finding is in agreement with results in Figure 1. Although the SEM results cannot show the EPS contents directly, the observation of more dense cell culture suggests that the production of EPS makes cells more clustered.

**Figure 4:**
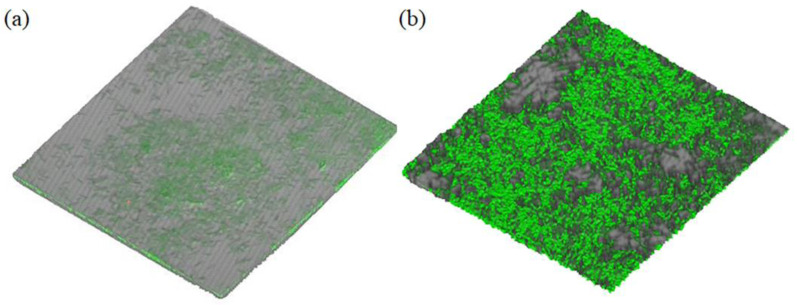
The structure of diatom-biofilm (*Cylindrotheca* sp.) effected by bacterial quorum-sensing signal molecule C12-HSL with CLSM analysis. (**a**) Control; (**b**) C12-HSL.

However, the different AHL-treated groups show no significant changes. 3D images of the diatom-biofilm have been observed in CLSM. The bacterial signal molecule C12-HSL significantly helps the formation of the diatom-biofilm, which is shown as a model in Figure 4.

The results of the thickness and fluorescence intensity of the diatom-biofilm are listed in Table 2. The biofilm thickness increases when the diatoms are exposed to AHLs (C8-HSL and C12-HSL) and the fluorescence intensities are also enhanced. The intensity values of the treatment groups using C4-HSL, C8-HSL, and C12-HSL are nearly 35.9 times, 36.7 times, and 48.6 times of the control.

**Figure 5:**
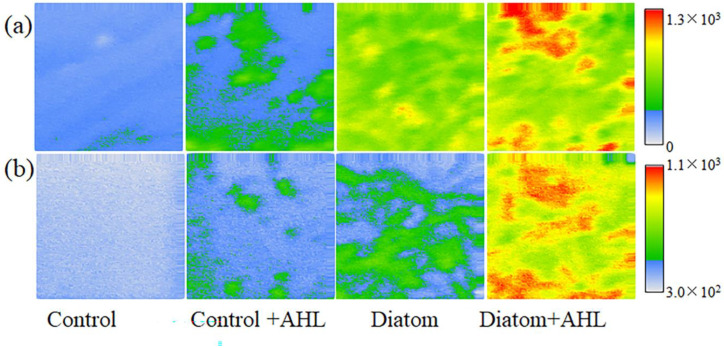
2D ToF-SIMS images of diatom-biofilm (*Cylindrotheca* sp.) affected by bacterial quorum-sensing signal molecule C12-HSL in the (**a**) negative mode and (**b**) positive mode. The analyzed area is 500 μm by 500 μm.

Figure 5 depicts the 2D image comparison of total ions intensity, which represents the EPS of diatom cells through ToF-SIMS analysis. The results show that the EPS content of the diatom cells increase with the treatment of C12-HSL in both the negative and postive modes. The control of f/2 medium contains less EPS content as expected. The additon of C12-HSL shows some EPS distribution in the 2D image, and the diatom cells treated with C12-HSL show the highest ion intensities in the 2D image compared with other groups including the group of diatom cells without C12-HSL in both the negative and positive modes.

## 4. Discussion

There has been a recent shift in our understanding of bacterial QS signal molecules, from bacterial cross-talk to prokaryote-eukyarote interactions. Although QS signals have been widely considered as regulators of prokaryotic gene expression, they can also modify the behavior of fungal, plant, and animal cells. For example, 3-oxo-C12-HSL can induce apoptosis in various cell types via a mechanism that could be blocked by inhibitors of calcium signaling [36,37]. As to animal cells, AHLs with an 11–13 carbon side chain length and a 3-oxo- or 3-hydroxy- substituent functional group exhibited a potent immunomodulatory activity and AHLs could be used as an indicator for site selection of permanent attachement of *Balanus improvisus* cyprid [27]. In addition, many different plant-associated bacteria possessed a protein closely related to the QS LuxR family protein that binded and responded to plant compounds, and the protein was involved in the communication between plant and bacteria, such as *Rhizobia*, *Xanthomonas*, and *Pseudomonas* [38,39]. All these reports show that AHLs are crucial molecules that regulate the interaction of prokaryote and eukaryote. In the previous study, the C10-HSL, 3-OXO-C10-HSL and 3-OH-C10-HSL also have been verified to increase the biofilm formation of *Cylindrotheca* sp. by analyzing physiological data [40]. In this study, C4-HSL, C8-HSL, and C12-HSL of AHLs were also found to stimulate the biofilm by directly observing the diatom-biofilm composition.

The close relationships between bacteria and algae have been investigated and reported [6,41]. However, very few reports have studied this topic in the fouling biofilm, especially the relevance of bacteria for diatom adhesion in biofilms. It was found that the growth and attachement of diatoms was driven by bacteria [42,43], but it is still unkown whether the interaction of bacteria and diatoms in fouling biofilms is mediated by AHLs. This has been the first study, to the best of our knowledge, to demonstrate that bacteria-produced QS signal molecule AHLs, including C4-HSL, C8-HSL, and C12-HSL, facilitate the formation of the diatom-biofilm *Cylindrotheca* sp. Furthermore, these effects depend on the exposure concentrations of AHLs and the length of the carbon chains of AHLs. Generally speaking, Chl.a contents and algal cell numbers are used to represent the biomass of algae. Our analysis shows that the Chl.a content in the diatom *Cylindrotheca* sp. increases when raising the exposure concentrations of AHLs, and algal cell numbers increase corresponding to the length of carbon chains at the designed exposure of AHLs. These results suggest that AHLs aid the growth of the diatom-biofilm.

Equally important, EPS, as the main components of the diatom-biofilm, is mainly comprised of PS and PN, which form an adhesive mucilage and allow them to build and hold the biofilm together [43]. In this study, EPS results show that AHLs assist the secretion of EPS in the diatom *Cylindrotheca* sp., especially PS, and the effects of AHLs on the diatom depend on the types of signal molecules. The different kinds of AHLs induce distinctive reflections of the diatom-biofilm. For example, C4-HSL at lower concentrations decreases the PS and total EPS content in the diatom-biofilm, which is opposite to the effect of Chl.a. It seems that C4-HSL improves the Chl.a synthesis, yet decreases the PS contents in the diatom-biofilm. Both C8-HSL and C12-HSL at higher concentrations increase the total EPS content. However, C8-HSL only promotes the secretion of PS and C12-HSL decreases the PN content and increase the PS content in the diatom-biofilm. It is speculated that the action mechanisms of AHLs are different from the EPS secretion of the diatom [40]. Although the PN and PS contents in the diatom-biofilm vary on the basis of different structures and concentrations of AHLs, the total EPS contents are significantly increased by the three kinds of AHLs when the exposure concentration reaches 10 mg/L. These results further show the importance of AHLs on the formation of the diatom-biofilm.

In addition, AHLs increase the ratio of EPS to Chl.a (EPS/Chl.a) except C8-HSL, which indicates that the other two kinds of AHLs could elevate the secretion of EPS in the diatom. For C8-HSL, the increase in the EPS content is accompanied by no change of the EPS/Chl.a ratio, and this might be attributed to the proliferation of the diatom cells. It was reported that the ratio of PN and PS might be closely related to EPS adherence and the lower PN/PS ratio implied the higher adherence of EPS [44,45]. Our present results show that C8-HSL and C12-HSL significantly decrease the values of PN/PS, which suggests that the two kinds of signal molecules enhance the adherence of the diatom and promote biofilm formation. Finally, the morphology of the diatom-biofilm of *Cylindrotheca* sp. determined by alcian blue staining and SEM imaging demonstrates that AHLs faciliate biofilm formation and increase the algal density with the increased length of carbon chains. CLSM suggests that the long carbon chains of AHLs (C8-HSL and C12-HSL) increase the thickness of the biofilm and the fluorescence intensities are significantly enhanced with the longer carbon chains of AHLs. ToF-SIMS has been first used to directly show EPS distributions in the diatom-biofilm to the best of our knowledge in this work. Many proteins, fatty acids, carbohydrates, and lipids are detected in the positive modes in biological biofilm samples, and most fatty acids, lipids, and nucleic acids are found in the negative mode in ToF-SIMS [22,25,33,34,35,46,47]. All these organic molecules are components of the EPS of the diatom-biofilm. Our SIMS results verify that bacteria QS signal C12-HSL promotes the EPS produciton of diatom cells. All these results shown in 2D images are consistent with the values of Chl.a, attached algal cell numbers, and EPS contents, suggesting that AHLs are an important factor to influence the formation of the diatom-biofilm *Cylindrotheca* sp.

The mechanism of AHLs on diatom-biofilm formation deserves more studies in the future, especially concerning the biofilm gene expression. However, at present there are not any reports on the influence of the mechanism of AHLs in the biofilm formation of diatoms and other organisms, especially concerning the molecular mechanism. However, similar phenomena that AHLs promote the attachment of fouling algae and animals were found. For example, the attachment of *Euteromorpha* zoospores and of the cyprids of *Balanus improvisus* was first reported to be induced and enhanced by AHL signal molecules produced by bacteria in biofouling [27]. The numbers of attached zoospores and cyprids were closely correlated to the types and exposure concentrations. The longer carbon chains and the higher concentrations of AHLs were more beneficial to the attachment of the spores and cyprids. The zoospores were considered not to attach to the substrate if AHL-genes of bacteria were knocked out. In our previous report [40], we have found that the AHLs of 10 carbons with different carbon-chain structure facilitated the formation of diatom-biofilm (*Cylindrotheca* sp.) in combination with Ca^2+^ efflux, which might reduce the population motility and encourage the settlement of marine benthic diatoms. Similarly, this new study further demonstrates that AHLs promote the formation of the diatom-biofilm *Cylindrotheca* sp., and the effects depend on the type and exposure concentrations of AHLs. Thus, AHLs could play an important role in the attachment of fouling organisms and formation of fouling communities. We postulate that many kinds of AHLs are favorable to biofilm formation of diatoms due to EPS-facilitated production or reduced cell motility for settlement. This topic about EPS or motlity gene expression of diatom cells is worthing exploring in our future work.

## 5. Conclusions

A series of experiments were performed using multiple imaging techiques including CLSM, SEM, and ToF-SIMS and supplemented with NMR and GC-MS. The results described here show that bacterial QS signal molecules AHLs increase the attached biomass of diatoms, the EPS content, and the thickness of diatom-biofilms. The effects depend on the length of the carbon chains and the exposure concentrations of AHLs. These findings confirmed with advanced chemical imaging techniques suggest that AHLs boost the formation of the diatom-biofilm. Such molecular level understanding will improve our understanding of fouling biofilm formation and eventually prevent or reduce biofouling in the marine environment.

## Figures and Tables

**Table 1:** Effects of AHLs on the values of EPS/Chl.a and PN/PS in the diatom-biofilm (*Cylindrotheca* sp.).

Sample	Control	C4-HSL	C8-HSL	C12-HSL
EPS/Chl.a	1.0 ± 0.05	1.32 ± 0.1 *	0.94 ± 0.09	2.4 ± 0.39 **
PN/PS	1.0 ± 0.17	0.73 ± 0.03	0.53 ± 0.01 **	0.05 ± 0.01 **

* The values of the control groups are considered as 1.0 ± SE, and the treatment groups are presented as relative values for the convenient comparison. * (*p* < 0.05) and ** (*p* < 0.01) indicate significant differences compared with the corresponding control without AHLs. EPS represents extracellular polymeric substances, Chl.a is Chlorophyll a, PN is protein, and PS is polysaccharide.

**Table 2:** The thickness and the fluorescence intensity of the diatom-biofilm (*Cylindrotheca* sp.) effected by AHLs.

Group	Control	C4-HSL	C8-HSL	C12-HSL
Biofilm thickness (μm)	27.7 ± 4.04	28 ± 4.36	34.5 ± 3.54 *	35.5 ± 2.12 *
Fluorescence intensity	18.9 ± 18.37	678.9 ± 181.04	583.2 ± 118.06 **	918.3 ± 158.15 **

* (*p* < 0.05) and ** (*p* < 0.01) indicate significant differences compared with the corresponding control without AHLs.

## Data Availability

The data presented in this study are available on request from the corresponding author.

## Supplementary Materials

The following supporting information can be downloaded at: https://www.mdpi.com/article/10.3390/microorganisms11071841/s1, Figure S1: NMR and GC-MS spectra of bacterial quorum-sensing signal molecules acyl homoserine lactones (AHLs). The purity of (a) C4-HSL, (b) C8-HSL, and (c) C12-HSL are 95%, 98%, and 100%, respectively. Table S1. The f/2 medium components.

## References

[1] El-saied H.A.-A., Ibrahim A.M. (2020). Effective fabrication and characterization of eco-friendly nano chitosan capped zinc oxide nanoparticles for effective marine fouling inhibition. J. Environ. Chem. Eng..

[2] Rajitha K., Nancharaiah Y.V., Venugopalan V.P. (2020). Insight into bacterial biofilm-barnacle larvae interactions for environmentally benign antifouling strategies. Int. Biodeterior. Biodegrad..

[3] Rajitha K., Nancharaiah Y.V., Venugopalan V.P. (2020). Role of bacterial biofilms and their eps on settlement of barnacle (amphibalanus reticulatus) larvae. Int. Biodeterior. Biodegrad..

[4] Najid N., Hakizimana J.N., Kouzbour S., Gourich B., Ruiz-García A., Vial C., Stiriba Y., Semiat R. (2022). Fouling control and modeling in reverse osmosis for seawater desalination: A review. Comput. Chem. Eng..

[5] Qian P.-Y., Cheng A., Wang R., Zhang R. (2022). Marine biofilms: Diversity, interactions and biofouling. Nat. Rev. Microbiol..

[6] Lépinay A., Turpin V., Mondeguer F., Grandet-Marchant Q., Capiaux H., Baron R., Lebeau T. (2018). First insight on interactions between bacteria and the marine diatom haslea ostrearia: Algal growth and metabolomic fingerprinting. Algal Res..

[7] Paterson D.M., Hope J.A. (2021). Diatom biofilms: Ecosystem engineering and niche construction. Diatom Gliding Motility.

[8] Lukáčová A., Beck T., Koptašiková L., Benda A., Tomečková L., Trniková M., Lihanová D., Steiner J.M., Krajčovič J., Vesteg M. (2022). Euglena gracilis can grow in the mixed culture containing cladosporium westerdijkiae, lysinibacillus boronitolerans and pseudobacillus badius without the addition of vitamins b1 and b12. J. Biotechnol..

[9] Nowruzi B., Shishir A.M., Porzani J.S., Ferdous T.U. (2022). Exploring the interactions between algae and bacteria. Mini-Rev. Med. Chem..

[10] Shi H.-X., Wang J., Liu S.-Y., Guo J.-S., Fang F., Chen Y.-P., Yan P. (2023). Potential role of ahl-mediated quorum sensing in inducing non-filamentous sludge bulking under high organic loading. Chem. Eng. J..

[11] Vadakkan K., Choudhury A.A., Gunasekaran R., Hemapriya J., Vijayanand S. (2018). Quorum sensing intervened bacterial signaling: Pursuit of its cognizance and repression. J. Genet. Eng. Biotechnol..

[12] Huang Y.-L., Ki J.-S., Lee O.O., Qian P.-Y. (2009). Evidence for the dynamics of acyl homoserine lactone and ahl-producing bacteria during subtidal biofilm formation. ISME J..

[13] Billot R., Plener L., Grizard D., Elias M.H., Chabrière É., Daudé D. (2022). Applying molecular and phenotypic screening assays to identify efficient quorum quenching lactonases. Enzym. Microb. Technol..

[14] Mukherjee S., Bassler B.L. (2019). Bacterial quorum sensing in complex and dynamically changing environments. Nat. Rev. Microbiol..

[15] Anandan K., Vittal R.R. (2019). Quorum quenching activity of aiia lactonase kmmi17 from endophytic bacillus thuringiensis kmcl07 on ahl- mediated pathogenic phenotype in pseudomonas aeruginosa. Microb. Pathog..

[16] Tang X., Guo Y., Zhu T., Tao H., Liu S. (2019). Identification of quorum sensing signal ahls synthases in candidatus jettenia caeni and their roles in anammox activity. Chemosphere.

[17] Joint I., Tait K., Callow M.E., Callow J.A., Milton D., Williams P., Ca’mara M. (2002). Cell-to-cell communication across the prokaryote-eukaryote boundary. Science.

[18] Zhou D., Zhang C., Fu L., Xu L., Cui X., Li Q., Crittenden J.C. (2017). Responses of the microalga chlorophyta sp. To bacterial quorum sensing molecules (n-acylhomoserine lactones): Aromatic protein-induced self-aggregation. Environ. Sci. Technol..

[19] Yu X.-Y., Liu B., Yang L. (2013). Imaging liquids using microfluidic cells. Microfluid. Nanofluidics.

[20] Yu X.-Y. (2020). In situ, in vivo, and in operando imaging and spectroscopy of liquids using microfluidics in vacuum. J. Vac. Sci. Technol. A.

[21] Yang C., Wei W., Liu F., Yu X.-Y. (2019). Peak selection matters in principal component analysis: A case study of syntrophic microbes. Biointerphases.

[22] Wei W., Zhang Y., Komorek R., Plymale A., Yu R., Wang B., Zhu Z., Liu F., Yu X.-Y. (2017). Characterization of syntrophic geobacter communities using tof-sims. Biointerphases.

[23] Sui X., Zhou Y., Zhang F., Zhang Y., Chen J., Zhu Z., Yu X.-Y. (2018). Tof-sims characterization of glyoxal surface oxidation products by hydrogen peroxide: A comparison between dry and liquid samples. Surf. Interface Anal..

[24] Fu Y., Zhang Y., Zhang F., Chen J., Zhu Z., Yu X.-Y. (2018). Does interfacial photochemistry play a role in the photolysis of pyruvic acid in water?. Atmos. Environ..

[25] Ding Y., Zhou Y., Yao J., Szymanski C., Frdrickson J., Shi L., Cao B., Zhu Z., Yu X.-Y. (2016). In situ molecular imaging of the biofilm and its matrix. Anal. Chem..

[26] Guillard R.R.L., Ryther J.H. (1962). Studies of marine planktonic diatoms: I. Cyclotella nana hustedt, and detonula confervacea (cleve) gran. Can. J. Microbiol..

[27] Tait K., Havenhand J. (2013). Investigating a possible role for the bacterial signal molecules n-acylhomoserine lactones in balanus improvisus cyprid settlement. Mol. Ecol..

[28] Yang C., Zhou J., Liu S., Fan P., Wang W., Xia C. (2013). Allelochemical induces growth and photosynthesis inhibition, oxidative damage in marine diatom phaeodactylum tricornutum. J. Exp. Mar. Biol. Ecol..

[29] Cohen L., Walt D.R. (2019). Highly sensitive and multiplexed protein measurements. Chem. Rev..

[30] Masuko T., Minami A., Iwasaki N., Majima T., Nishimura S., Lee Y.C. (2005). Carbohydrate analysis by a phenol-sulfuric acid method in microplate format. Anal. Biochem..

[31] Zgłobicka I., Gluch J., Liao Z., Werner S., Guttmann P., Li Q., Bazarnik P., Plocinski T., Witkowski A., Kurzydlowski K.J. (2021). Insight into diatom frustule structures using various imaging techniques. Sci. Rep..

[32] Zhou S., Bu L., Shi Z., Deng L., Zhu S., Gao N. (2018). Electrochemical inactivation of microcystis aeruginosa using bdd electrodes: Kinetic modeling of microcystins release and degradation. J. Hazard. Mater..

[33] Zhang Y., Komorek R., Son J., Riechers S., Zhu Z., Jansson J., Jansson C., Yu X.-Y. (2021). Molecular imaging of plant–microbe interactions on the brachypodium seed surface. Analyst.

[34] Zhang Y., Komorek R., Zhu Z., Huang Q., Chen W., Jansson J., Jansson C., Yu X.-Y. (2022). Mass spectral imaging showing the plant growth-promoting rhizobacteria’s effect on the brachypodium awn. Biointerphases.

[35] Ding Y., Zhou Y., Yao J., Xiong Y., Zhu Z., Yu X.-Y. (2019). Molecular evidence of a toxic effect on a biofilm and its matrix. Analyst.

[36] Grainha T., Jorge P., Alves D., Lopes S.P., Pereira M.O. (2020). Unraveling pseudomonas aeruginosa and candida albicans communication in coinfection scenarios: Insights through network analysis. Front. Cell. Infect. Microbiol..

[37] Lu X., Wang Y., Chen C., Feng Z., Huo Y., Zhou D. (2022). C12-hsl is an across-boundary signal molecule that could alleviate fungi galactomyces’s filamentation: A new mechanism on activated sludge bulking. Environ. Res..

[38] Gonza´lez J.F., Venturi V. (2013). A novel widespread interkingdom signaling circuit. Trends Plant Sci..

[39] Cellini A., Buriani G., Correia C., Fiorentini L., Vandelle E., Polverari A., Santos C., Vanneste J.L., Spinelli F. (2022). Host-specific signal perception by psar2 luxr solo induces pseudomonas syringae pv. Actinidiae virulence traits. Microbiol. Res..

[40] Yang C., Fang S., Chen D., Wang J.-H., Liu F., Xia C. (2016). The possible role of bacterial signal molecules n-acyl homoserine lactones in the formation of diatom-biofilm (*Cylindrotheca* sp.). Mar. Pollut. Bull..

[41] Liu Q., Xuan F., Zhiya S., Wenxin S., Shuo W., Ji L. (2022). Enhanced wastewater treatment performance by understanding the interaction between algae and bacteria based on quorum sensing. Bioresour. Technol..

[42] Bruckner C.G., Rehm C., Grossart H.P., Kroth P.G. (2011). Growth and release of extracellular organic compounds by benthic diatoms depend on interactions with bacteria. Environ. Microbiol..

[43] Khan M.J., Singh R., Shewani K., Shukla P., Bhaskar P.V., Joshi K.B., Vinayak V. (2020). Exopolysaccharides directed embellishment of diatoms triggered on plastics and other marine litter. Sci. Rep..

[44] Sweity A., Ying W., Ali-Shtayeh M.S., Yang F., Bick A., Oron G., Herzberg M. (2011). Relation between eps adherence, viscoelastic properties, and mbr operation: Biofouling study with qcm-d. Water Res..

[45] El-Naggar N.E.-A., Hussein M.H., Shaaban-Dessuuki S.A., Dalal S.R. (2020). Production, extraction and characterization of chlorella vulgaris soluble polysaccharides and their applications in agnps biosynthesis and biostimulation of plant growth. Sci. Rep..

[46] Hua X., Marshall M.J., Xiong Y., Ma X., Zhou Y., Tucker A.E., Zhu Z., Liu S., Yu X.-Y. (2015). Two-dimensional and three-dimensional dynamic imaging of live biofilms in a microchannel by time-of-flight secondary ion mass spectrometry. Biomicrofluidics.

[47] Hua X., Yu X.-Y., Wang Z., Yang L., Liu B., Zhu Z., Tucker A.E., Chrisler W.B., Hill E.A., Thevuthasan T. (2014). In situ molecular imaging of a hydrated biofilm in a microfluidic reactor by tof-sims. Analyst.

