# Supplementary material for: Revealing the Bacterial Quorum-Sensing Effect on the Biofilm Formation of Diatom Cylindrotheca sp. Using Multimodal Imaging
Source: Microorganisms. 2023 Jul 20;11(7):1841. doi: 10.3390/microorganisms11071841 (PMC10383543; doi:10.3390/microorganisms11071841)

Revealing the Bacterial Quorum-Sensing Effect on the Biofilm Formation of Diatom *Cylindrotheca* sp. Using Multimodal Imaging

Cuiyun Yang [1,2,*], Guojuan Song [2,†], Jiyoung Son [3], Logan Howard [1] and Xiao-Ying Yu [1,*]

[1] Materials Science and Technology Division, Oak Ridge National Laboratory, Oak Ridge, TN 37830, USA
[2] Laboratory for Marine Ecology and Environmental Science, Qingdao National Laboratory for Marine Science and Technology, Qingdao 266071, China
[3] Energy and Environment Directorate, Pacific Northwest National Laboratory, Richland, WA 99354, USA
[*] Correspondence: yangcy29@163.com (C.Y.); yuxiaoying@ornl.gov (X.-Y.Y.); Tel.: +1-865-574-4628 (X.-Y.Y.)
[†] Current address: College of Chemistry & Chemical Engineering, Yantai University, Yantai 264005, China.

Supplemental file

Table S1. The f/2 medium components.

No.	Nutrient elements	Working solution（mg/L）
1	$NaNO_3$	75
2	NaH_2PO_4	5
3	Na_2EDTA	20
4	$FeCl_3 \cdot 6H_2O$	3.16
5	$CuSO_4 \cdot 5H_2O$	0.01
6	$ZnSO_4 \cdot 7H_2O$	0.023
7	$CoCl_2 \cdot 6H_2O$	0.012
8	$MnCl_2 \cdot 4H_2O$	0.18
9	$Na_2MoO_4 \cdot 2H_2O$	0.07
10	Thiamine HCl (V_{B1})	0.1 µg
11	Cyanocobalamin (V_{B12})	0.5 µg
12	Biotin	0.5 µg
13	Filtered seawater	1000 mL

Figure S1. NMR and GC-MS spectra of bacterial quorum sensing signal molecules acyl homoserine lactones (AHLs). The purity of (a) C4-HSL, (b) C8-HSL, and (c) C12-HSL are 95%, 98%, and 100%, respectively.

a: C4-HSL

^{1}H-NMR (500 MHz, $CDCl_3$) δ_H: 0.97 (3H, t, J = 7.4 Hz, $C\underline{H}_3$), 1.68 (2H, m, $CH_3C\underline{H}_2CH_2CO$), 2.17 (1H, m, $OCH_2C\underline{H}'HCHNH$), 2.25 (2H, td, J = 7.6, 1.3 Hz, $CH_3CH_2C\underline{H}_2CO$), 2.81 (1H, m, $OCH_2C\underline{H'}HCHNH$), 4.30 (1H, ddd, J = 11.2, 9.4, 6.0 Hz， $OC\underline{H}'HCH_2CHNH$), 4.47 (1H, td, J = 9.0, 0.7 Hz, $OCH'\underline{H}CH_2CHNH$), 4.60 (1H, ddd, J = 11.5, 8.6, 6.3 Hz, $OCH'HCH_2C\underline{H}NH$), 6.34 (1H, s, $N\underline{H}$); ^{13}C-NMR (125 MHz, $CDCl_3$) δ_C: 13.6 (CH_3), 18.9 ($CH_3\underline{C}H_2CH_2CO$), 30.3 ($OCH_2\underline{C}H_2CHNH$), 38.0 ($CH_3CH_2\underline{C}H_2CO$), 49.1 ($OCH_2CH_2\underline{C}HNH$), 66.1 ($O\underline{C}H_2CH_2CHNH$), 173.6 (C=O amide), 175.7 (C=O lactone)；MS(EI): *m/z* 172, 143, 125, 101, 85, 83, 56.

(1) NMR spectra

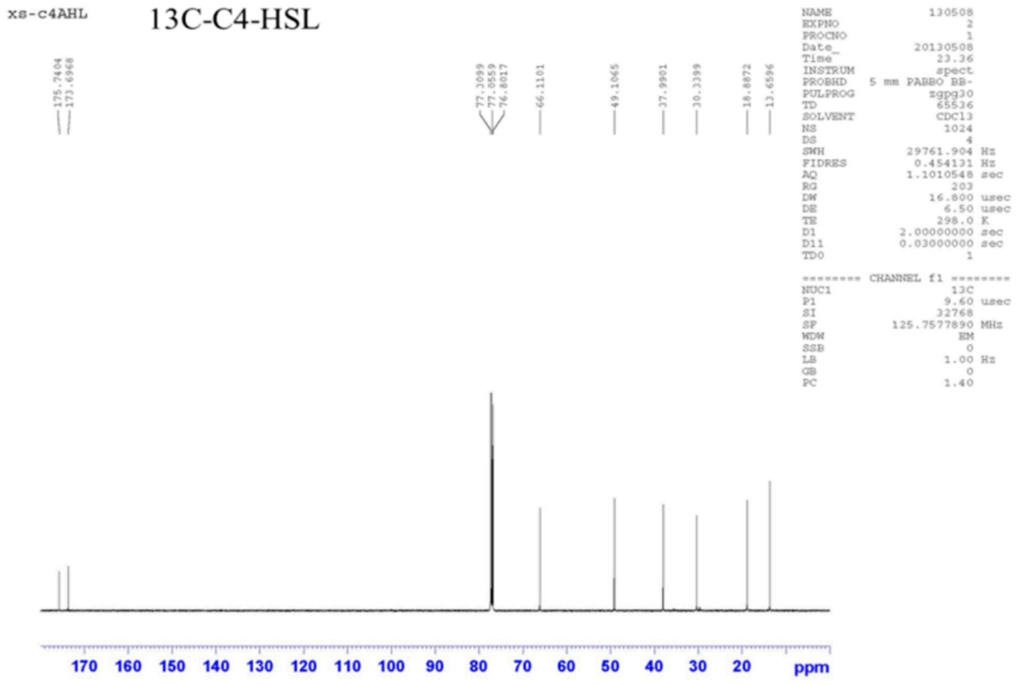

xs-c4AHL

1H-C4-HSL

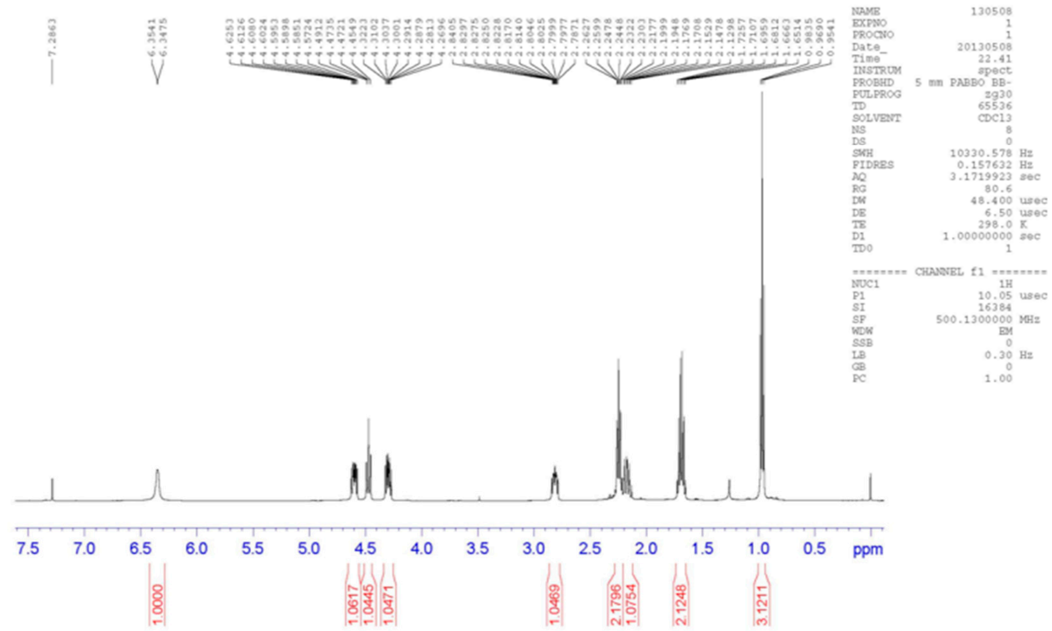

(2) GC-MS spectra

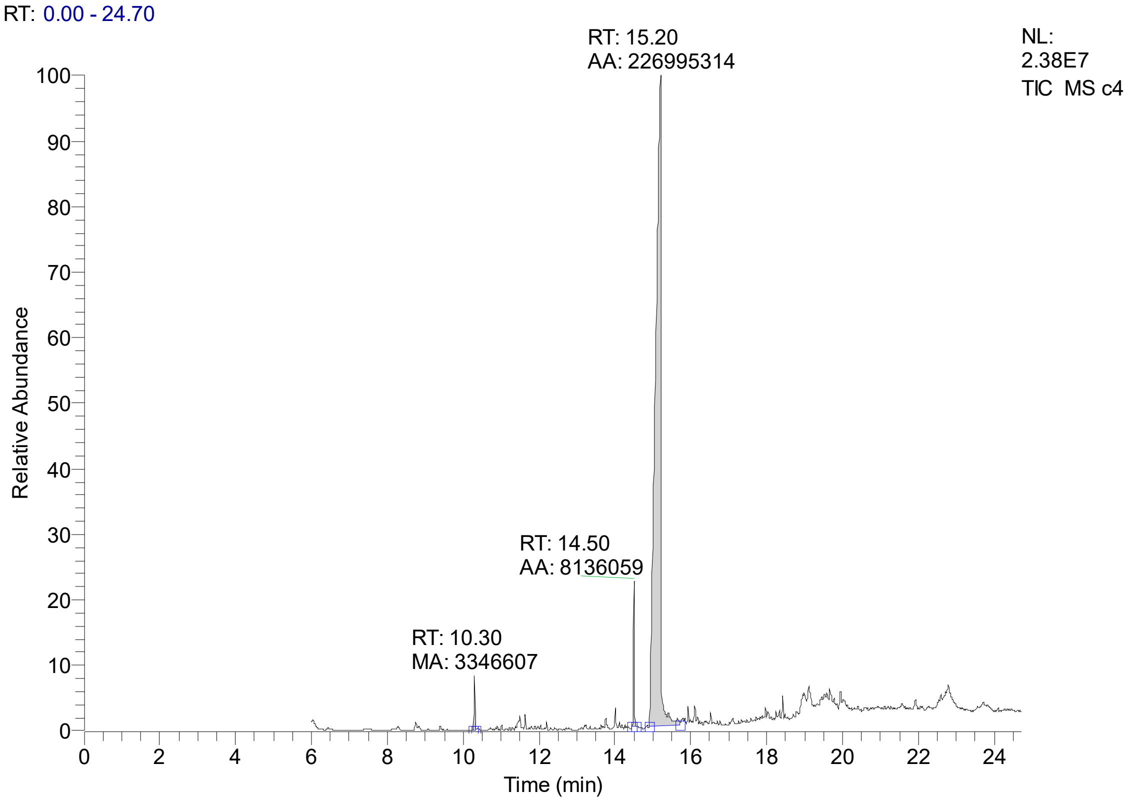

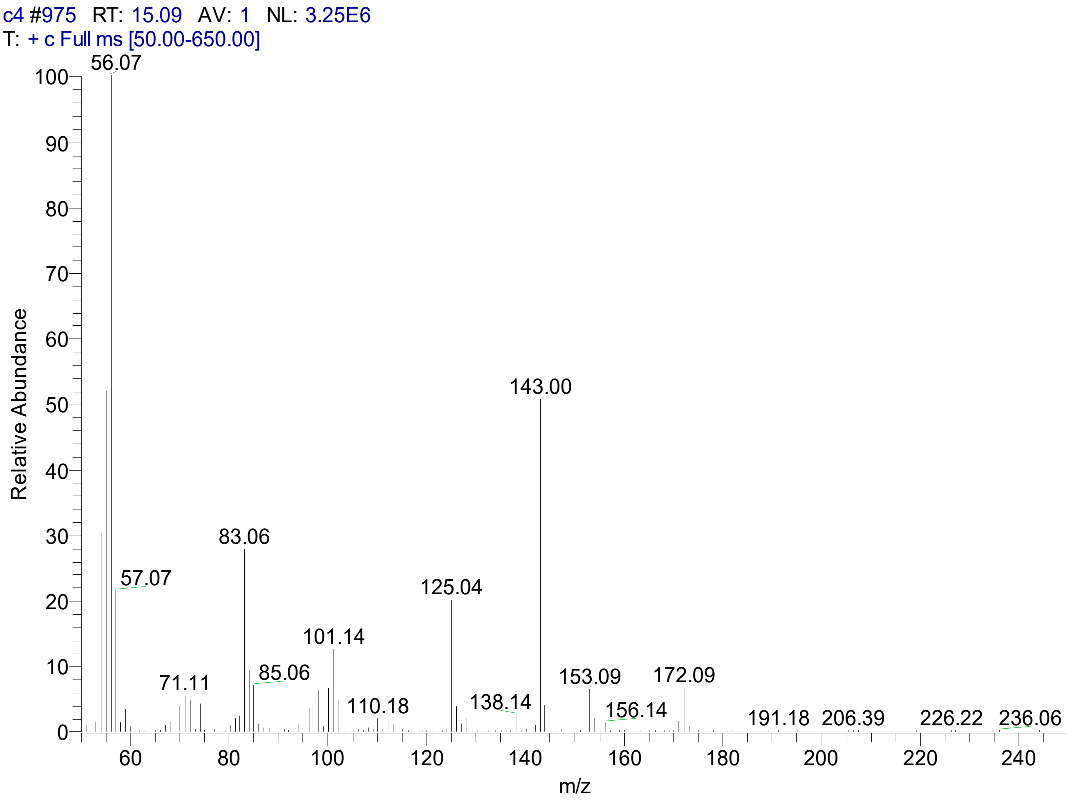

b: C8-HSL

^{1}H-NMR (500 MHz, $CDCl_3$) δ_H: 0.9 (3H, t, J = 6.5 Hz, CH_3), 1.21–1.43 (8H， m，$(CH_2)_4CH_3$）, 1.65–1.69 (2H, m, CH_2CH_2CO), 2.15 (1H, m, $OCH_2CH'HCHNH$), 2.24–2.28 (2H, m, CH_2CH_2CO), 2.87 (1H, m, $OCH_2CH'HCHNH$), 4.31 (1H, ddd, J = 11.0, 9.3, 6.1 Hz, $OCH'HCH_2CHNH$), 4.49 (1H, t, 9.0 Hz, $OCH'HCH_2CHNH$), 4.58 (1H, ddd, J = 11.5, 8.5, 5.9 Hz, OCH_2CH_2CHNH), 6.1 (1H, s, NH); ^{13}C-NMR (125 MHz, $CDCl_3$) δ_C: 14.0 (CH_3), 22.6 (CH_3CH_2), 25.4 ($CH_2(CH_2)_4CO$), 28.9 ($CH_2(CH_2)_3CO$), 29.1 ($CH_2(CH_2)_2CO$), 30.5 (CH_2CH_2CO), 31.6 (CH_2CH_2CO), 36.2 (OCH_2CH_2CHNH), 49.2 (OCH_2CH_2CHNH), 66.1 (OCH_2CH_2CHNH), 173.7 (C=O amide), 175.6 (C=O lactone)；MS(EI): *m/z* 228, 198, 156, 143, 124, 101, 83.

(1) NMR spectra

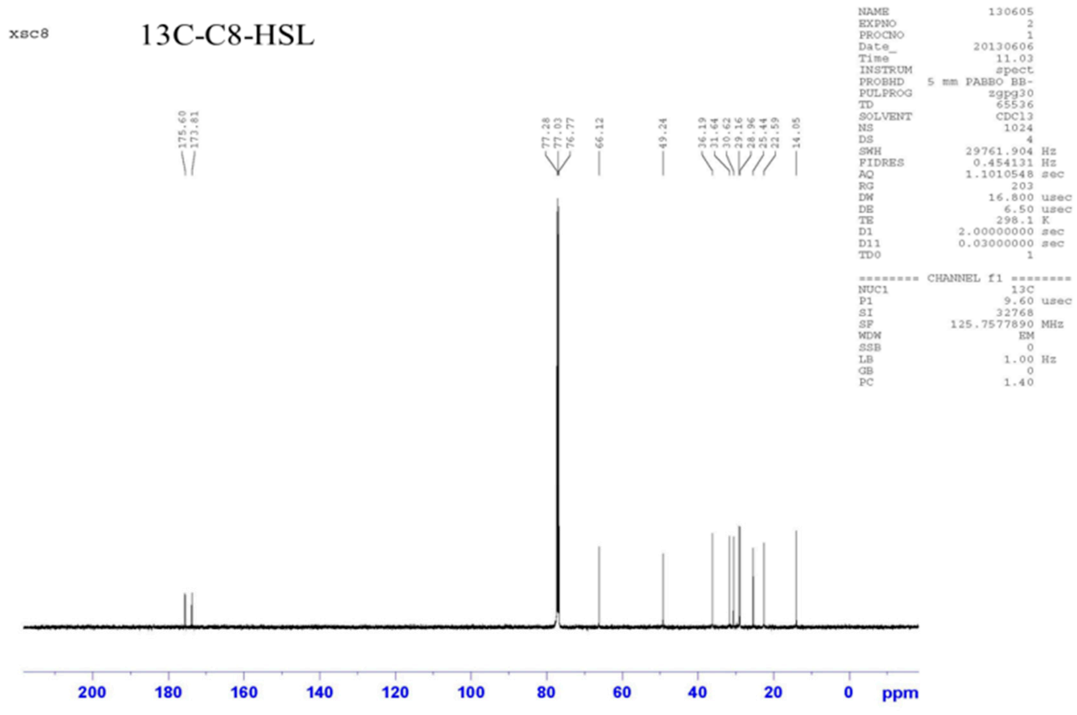

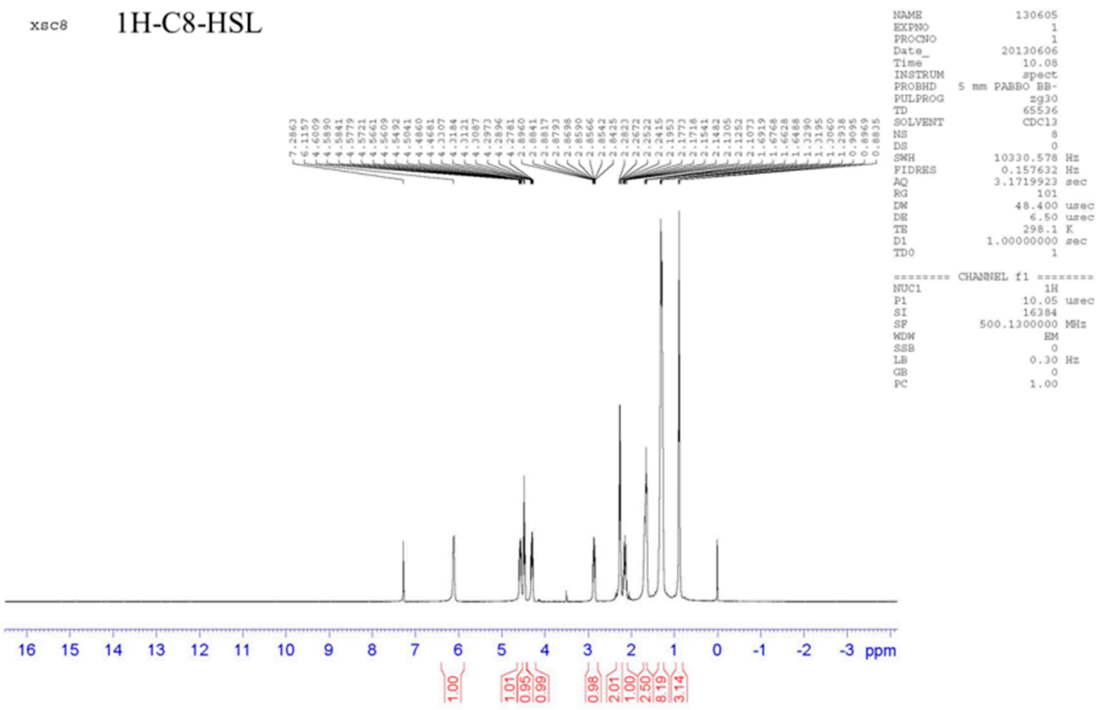

(2) GC-MS spectra

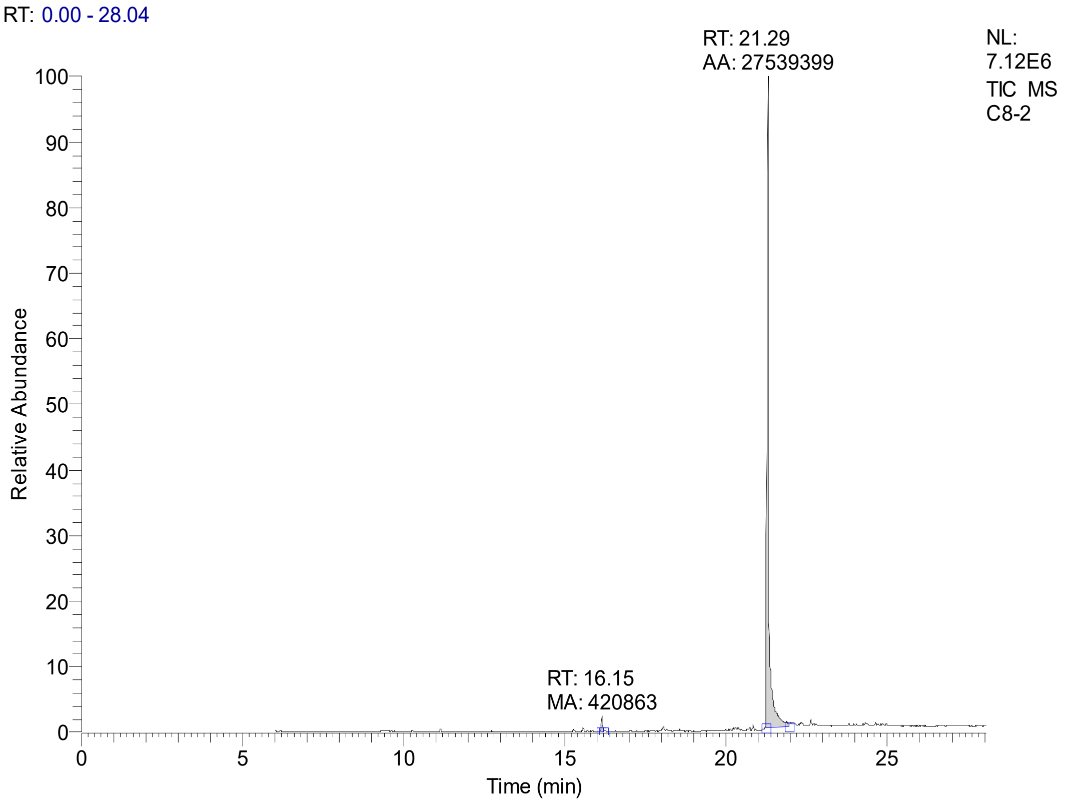

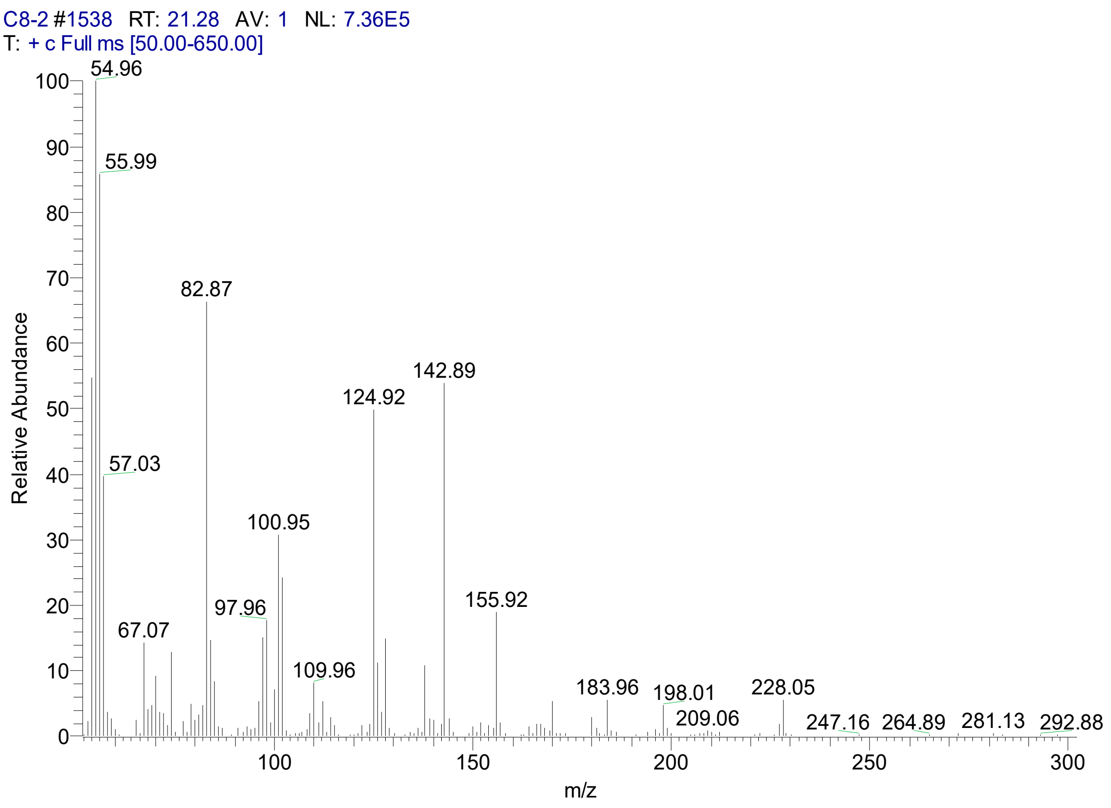
C8-2 #1538 RT: 21.28 AV: 1 NL: 7.36E5
T: + c Full ms [50.00-650.00]
Relative Abundance
m/z
54.96
55.99
57.03
67.07
82.87
97.96
100.95
109.96
124.92
142.89
155.92
183.96
198.01
209.06
228.05
247.16
264.89
281.13
292.88

c: C12-HSL

^{1}H-NMR (500 MHz, $CDCl_3$) δ_H: 0.89 (3H, t, J = 7.0 Hz, CH_3), 1.20–1.40 (16H, m, $CH_3(C\underline{H}_2)_8$), 1.60–1.69 (2H, m, $C\underline{H}_2CH_2CO$), 2.15 (1H, m, $OCH_2C\underline{H}'HCHNH$), 2.27 (2H, m, $CH_2C\underline{H}_2CO$), 2.87 (1H, m, $OCH_2CH'\underline{H}CHNH$), 4.30 (1H, ddd, J = 11.2, 9.3, 5.9 Hz, $OC\underline{H}'HCH_2CHNH$), 4.48 (1H, t, J = 9.1 Hz, $OCH'\underline{H}CH_2CHNH$), 4.58 (1H, ddd, J = 11.6, 8.6, 6.0 Hz, $OCH_2CH_2C\underline{H}NH$), 6.15 (1H, s, $N\underline{H}$); ^{13}C-NMR (125 MHz, $CDCl_3$) δ_C: 14.1 (CH_3), 22.7 (CH_2), 25.5 (CH_2), 29.2 (CH_2), 29.3 (CH_2), 29.3 (CH_2), 29.5 (CH_2), 29.5 (CH_2), 29.6 (CH_2), 30.6 (CH_2), 31.9 ($CH_2\underline{C}H_2CO$), 36.2 ($OCH_2\underline{C}H_2CHNH$), 49.2 ($OCH_2CH_2\underline{C}HNH$), 66.1 ($O\underline{C}H_2CH_2CHNH$), 173.8 (C=O amide), 175.63 (C=O lactone); MS(EI): *m/z* 284, 254, 240, 198, 170, 156, 143, 125, 102, 83, 57, 55.

(1) NMR spectra

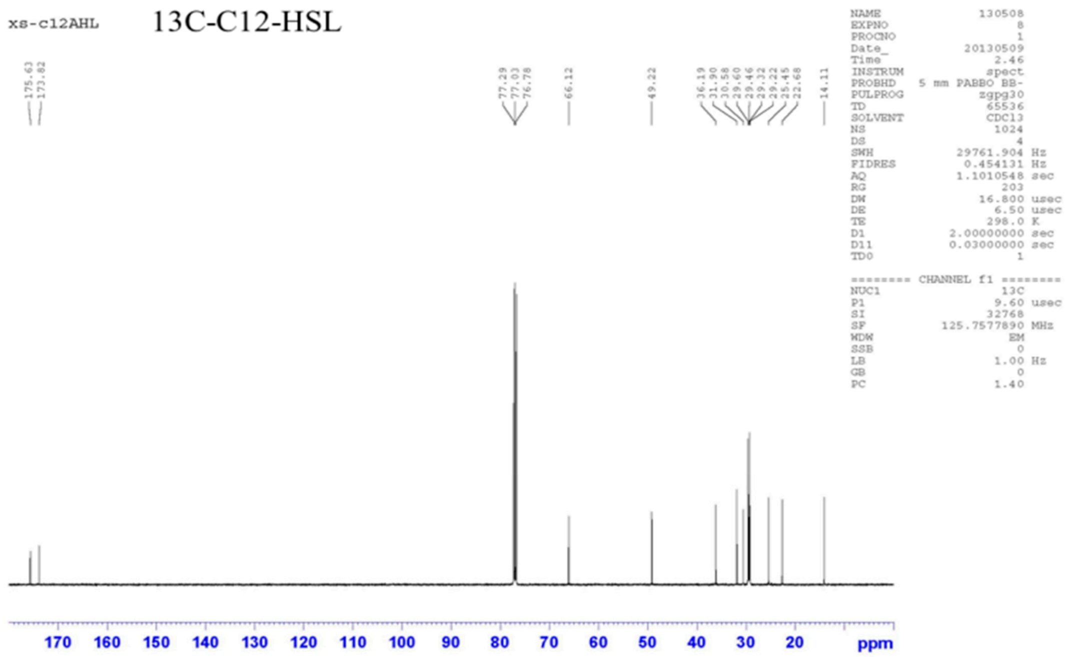

xs-c12AHL 1H-C12-HSL

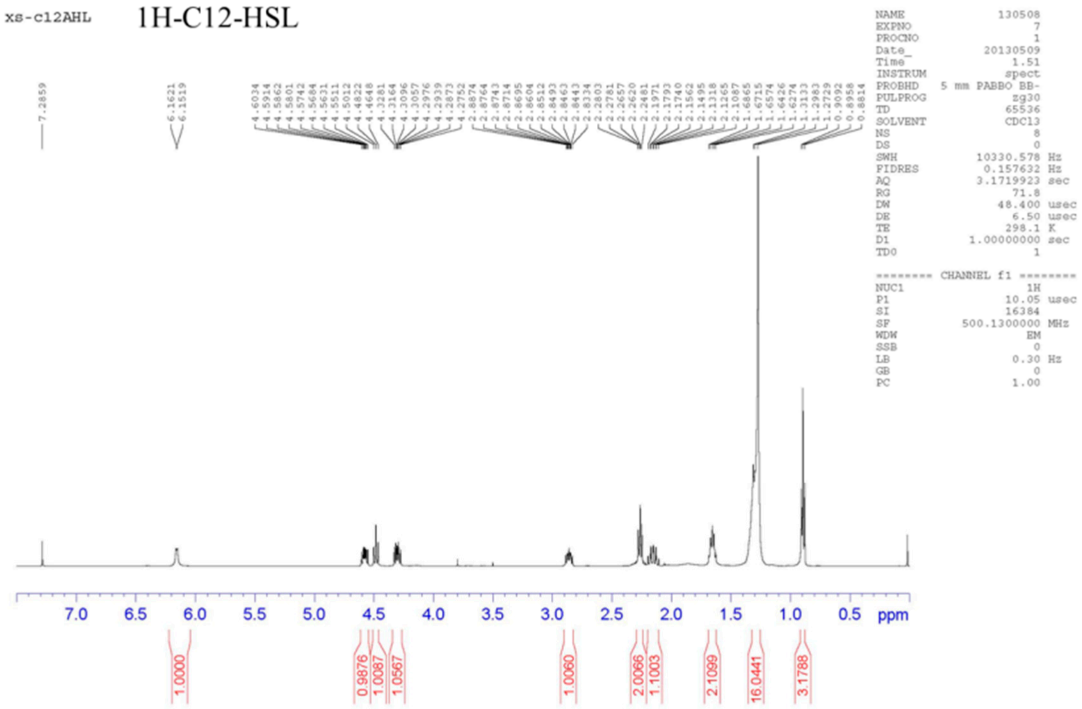

(2) GC-MS spectra

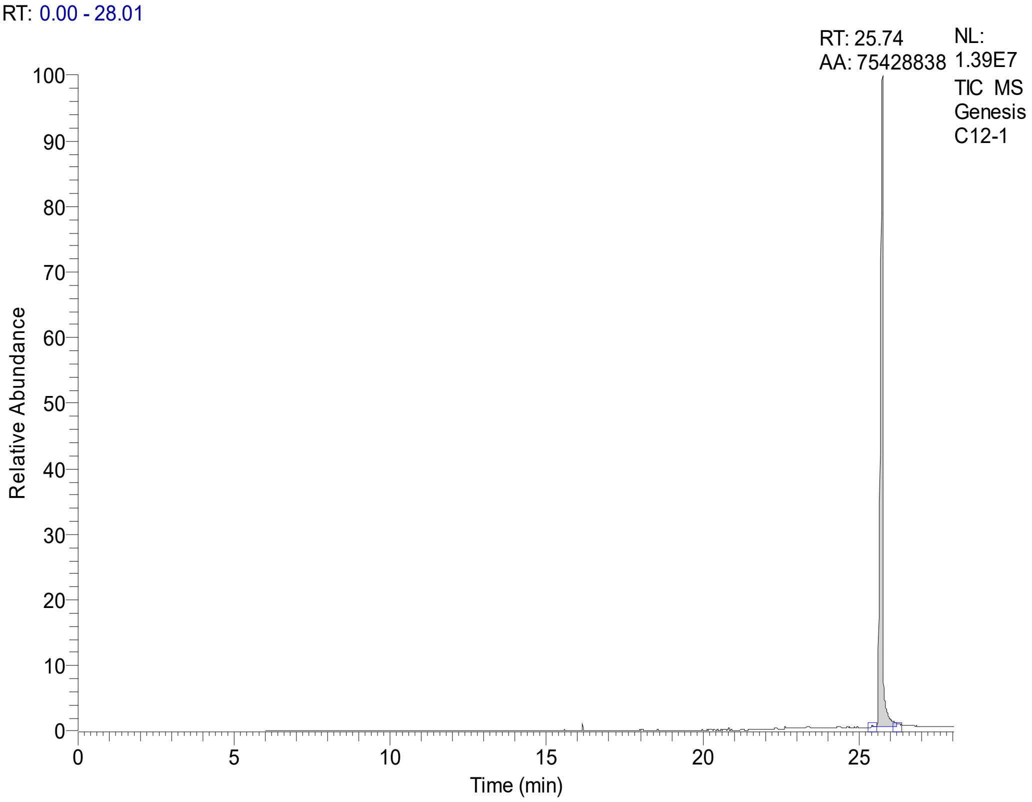

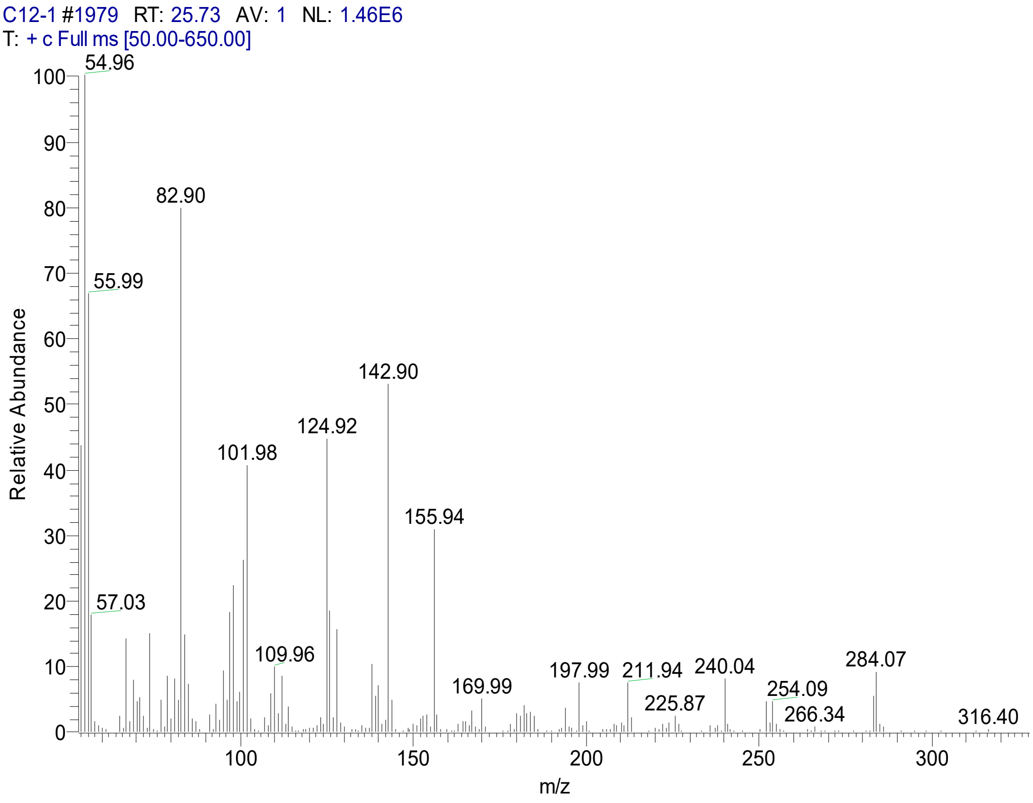

Supplement: Supplementary file 1 [file microorganisms-11-01841-s001.zip › microorganisms-2459141-supplementary.pdf]
